# AI-driven hybrid framework for enhanced pest detection and resource optimization using graph networks and deep reinforcement learning

**DOI:** 10.1038/s41598-026-47987-5

**Published:** 2026-04-19

**Authors:** Basu Dev Shivahare, Gambhir Singh, Rahat Naz, Suman Avdhesh Yadav, Sandeep Kumar Mathivanan, Sangeetha S.K.B

**Affiliations:** 1 School of Computing Science and Engineering, Galgotias University, Greater Noida, 203201 India; 2 GNIOT(Engg. Institute), Greater Noida, India; 3 School of Computer Science, University of Petroleum and Energy Studies (UPES), Dehradun, India; 4 School of Computer Science & Engineering, IILM University, Greater Noida, India; 5 Manipal Institute of Technology Bengaluru, Manipal Academy of Higher Education, Manipal, India; 6 Present Address: Department of Computer Science & Engineering- AI & ML, KG Reddy College of Engineering and Technology, Hyderabad,Telangana, 501504, India

**Keywords:** Precision agriculture, Graph convolutional networks, AutoML, Deep reinforcement learning, Pest detection, Resource optimization, Computational biology and bioinformatics, Ecology, Ecology, Environmental sciences, Mathematics and computing, Plant sciences

## Abstract

Farming is challenging due to climatic changes, insects, and inefficient resource management. Therefore, there is a need for smarter and autonomous systems. Traditional AI face challenges in solving problems including real-time optimization of resources, adaptation to environments, and fusion of data. In this study, a new framework is introduced that combines Graph Convolutional Networks (GCN), AutoML, and Deep Reinforcement Learning (DRL) to provide a change in precision agriculture. The system enhances resource management, pest detection, and crop immunity against diseases by considering space and time information. GCNs model the interaction of agricultural fields with environmental conditions, such as soil moisture, humidity, and temperature, both spatially and temporally. They monitor stages of crop development and pests. This spatial optimization allows dynamic real-time optimization. AutoML minimizes human expertise by automatically adapting parameters and structure across different areas and situations in agriculture. DRL drives an environment-adaptive decision-making process that automatically optimizes the control of resources and pests via adaptation based on environmental feedback. Two datasets were utilized to test the framework: the IoT Smart Farm Dataset (temperature, health of crops, soil moisture) and the Precision Agriculture Crop Dataset (pest infestation, satellite images, climatic data). Stability in yield improved by 21.7% (± 2.3%) compared to baseline strategies, accuracy in crop health assessment improved by 96.8%, and accuracy in the detection of pests improved by 95.3%, according to the results. The DRL technology saved 14.2% on fertilizer use and 16.4% on water usage. The approach provides efficient and scalable solutions for short-term and long-term agricultural challenges while setting a new benchmark for AI-farming and climate change resilience.

## Introduction

Agriculture is the backbone of world food provision but has become far more challenging with global climate change, pest infestation, and poor resource management. These factors are further heightened by the increasing demand for food, as agriculture needs to be more productive and sustainable. Traditional farming is generally labor-intensive, inefficient, and insensitive to changes in the environment. Recently, the adoption of state-of-the-art technologies like AI has been promising in offering solutions for the complex problems that face the modern agriculture sector. Resource optimization, crop health management, and even pest identification can be done through AI mechanisms.

The areas where current AI techniques lag include the integration of different forms of data, adaptation to contexts, and real-time optimization of resources. Traditional AI models, in the form of Machine Learning (ML) and Deep Learning (DL) techniques, form the backbone of precision agriculture in various operations related to yield estimation, crop classification, identification of pests, among others. Other areas where machine learning models have found effective applications include crop disease detection, soil classification, and pest infestation prediction, including decision trees, support vector machines, and random forests.

CNNs has processed sensor data and satellite images for identifying pests and tracking agriculture. Such models rely mostly on structured information, geographic dependencies among diverse agricultural regions, environmental factors, and crop health. They require a great amount of expert inputs and human tuning of hyperparameters, hence being far from scalable and responsive against diverse agricultural conditions. While conventional models are found to exhibit a number of deficiencies. Most conventional AI models do not consider the spatial-temporal and dynamic nature of agro-ecosystems.

These conditions, such as temperature, humidity, soil moisture, and insect pressure, are highly interrelated and vary decisively in space and time. Poor predictions and decisions because of neglect of this fact are common. Another major disadvantage is that most models involve a great deal of human involvement in feature extraction, data preprocessing, and adjustment of hyperparameters-requirements which are often time and resource-consuming for large-scale deployment across different agricultural regions characterized by different environmental conditions. Current AI models often cannot make adaptive, real-time decisions to react to every changing circumstance without human interference.

Static model-based pest management is unable to find new infestations or changes in the environment that require immediate attention. The following three AI techniques overcome these challenges: AutoML, Deep Reinforcement Learning, and Graph Convolutional Networks. This integrated framework is a completely autonomous system able to dynamically and in real time illustrate the complex relationships among various agricultural zones, environmental factors, and temporal trends. Thus, the integration of these approaches allows the proposed system to overcome challenges in normal AI techniques and provide an effective scalable solution to a number of challenges of modern agriculture.

The main contributions are.

1. To build an integrated system incorporating AutoML, Deep Reinforcement Learning, and Graph Convolutional Networks to advance pest identification, resource deployment, and spatial-temporal optimization in precision agriculture.
2. To create an independent Deep Reinforcement Learning-driven decision platform that constantly learns from environmental feedback to optimize resource allocation and pest management strategies without human intervention.
3. To create an AutoML-based system to tune the model’s architecture and hyperparameters automatically, so the framework can adjust to various agricultural regions and climatic conditions with limited human intervention.

Novel contributions are.

- AutoML-guided dynamic optimization of GCN architecture parameters during training.
- Joint GCN–DRL state fusion enabling graph-aware policy learning.
- Temporal graph embedding updates synchronized with DRL policy adaptation.
- Adaptive policy reconfiguration without manual hyperparameter tuning.

By combining GCNs, AutoML, and DRL, the system surpasses the limitations of traditional AI models to provide a scalable, self-sustaining means of crop health control, resource distribution, and pest treatment optimization. This system can revolutionize agriculture by enabling data-driven real-time decision-making that improves sustainability, productivity, and resilience to climate change. Section 2 discusses related study, Sect. 3 explains the system methodology, Sect. 4 demonstrates the experimental results, Sect. 5 concludes the study.

## Related study

AI and ML technologies have substituted the conventional practices of resource use, general productivity of crops, pest control, and disease detection in agriculture to a major extent. Most AI-based technologies are making disease detection, pest detection, and precision farming more autonomous. AI technologies, especially deep learning, reinforcement learning, and multimodal data fusion, have transformed many aspects of agricultural production and further enhanced the efficiency and sustainability of the sector.

Application of AI, mainly deep learning algorithms such as CNNs, has been promising in the detection of diseases and pests using satellite images, drone images, and other sensors. Such models of deep learning should identify initial signs of diseases or insect attacks to minimize loss in crops with quick responses. Deep learning models support real-time crop health monitoring and are most useful for evaluating visual data from remote sensing equipment^1^. The study shows that AI use in pest control can reduce pesticide application by a huge amount and increase crop yields. AI technology has now become very essential compared to conventional methods to achieve better identification and control of diseases and pests in crops^2^.

New integrated methods have been devised for improvement in diseases and pest diagnoses by researchers, including satellite information along with drone imagery, further supported by machine learning algorithms. The scalability and precision in systems for pest control have been enhanced by these kinds of methods through the fusion of data from more than one source. One such recent reinforcement learning-based system in drone technology is the SAN-GAT-RL for detection regarding diseases and pests, which has been very promising to enhance agricultural management under evolving conditions^3^. Remote sensing with AI-driven systems allowed for real-time monitoring of insects and ensured losses to crops are minimal by facilitating early interventions^4^.

AI technologies have gone a long way in the optimization of farm resources such as water, pesticide, and fertilizer^5^. Application of fertilizer and optimization of irrigation schedules were performed to avoid wastage by the use of RL^6^. Real-time weather monitoring, soil moisture, and other environmental factors enable more accurate utilization of resources by the AI systems that monitor those parameters. Reinforcement learning in combination with genetic algorithms turned out to be very effective when it was tried in optimizing resource use. These machine learning algorithms ensure dynamic realignment of agricultural practices to make optimum utilization of available resources^7,8^.

Precision agriculture that has very accurate information about crops and soil with a view to limiting environmental impacts while maximizing yield^9,10^. AI has its ability for the analysis of huge volumes of sensor, satellite, and Internet of Things sensor data, enabling better management of pests during resource delivery in a sustainable way at low costs^11^. Yield forecasting is increasingly being done using AI and machine learning algorithms. Such models take input data such as crop genetics, climatic conditions, and soil health to predict the yield of farm activities^12^.

Accurate yield forecasting is important in harvest planning, managing market expectations, and optimizing planting protocols^13^. Deep learning and XGBoost machine learning algorithms have scored well in agricultural production forecasting based on a range of considerations like plant health, pest infestation, and weather conditions^14^. Crop yield prediction software can locate parts of a field that may require special attention^15^. Farmers are now in a position to make better decisions regarding fertilization, irrigation, and pest control with the use of AI-based systems since these provide real-time monitoring of crop growth^16^.

AI has made it possible, and it is the era of smart farming where data still dictates agriculture^17^. Through precision farming, the farmer can monitor and manage the farm in a better way with the help of AI integrated with IoT, drones, and big data analysis. It allows for per-plant management through AI-powered systems tracking each plant for particular needs, be it irrigation, fertilization, or pest management^18^. These technologies have benefited agricultural producers by increasing production while reducing inputs and costs. For example, deep hybrid models combining machine learning with UAV-based IoT sensors have been used for identifying pests and forecasting crop diseases, hence enhancing the sustainability of the agricultural systems^19^.

Application of such technologies will empower farmers with effective and timely responses because of real-time observation and adaptive decision-making^20^. Farmers are accorded critical information concerning the management of crops, irrigation, and pest control by the decision support systems making use of AI. Because new challenges emanating from environmental change face the sector, AI is critical to climate-smart agriculture^21^. Machine learning models have improved the deterring factors for unfavorable climatic conditions on the crops, efficiency in resource utilization, and weather forecast^22^.

AI systems, comparing data from sources such as satellite images and weather forecasts can predict and suggest ways in which farmers can cope with changed climates. Research has shown that AI systems are most apt in handling water utilization in drought conditions^23^. Farmers are able to predict the irrigation needs and prevent the exploitation of water resources if AI is integrated with climatic data^24^. Moreover, AI is able to lead sustainable agriculture by promoting ecologically viable agricultural methods and reducing dependence on chemical inputs^25,26^.

Despite AI’s many advantages, there are weaknesses that need to be solved. Some of the key limitations towards pervasive use of AI technology in agriculture pertain to interpretability of machine learning models, scalability of the AI systems in rural settings, and data privacy. The development of AI models for geographic and climatic conditions is also required for effective deployment. This further calls for continued research in domain-specific AI solutions that can provide customized solutions for a range of agricultural environments^27,28^.

**Table 1:** Existing systems Vs Proposed system.

Aspect	Proposed System	Traditional Methods
Data usage	Leverages large, high-quality datasets from Kaggle (images of crops, pests, and diseases), which improves generalization and accuracy.	Limited by smaller, manually curated datasets or reliance on expert knowledge for feature extraction.
Feature extraction	Automatically learns hierarchical features (textures, shapes, and patterns) from raw data using convolutional layers.	Relies on handcrafted features, which may miss subtle patterns and interactions in the data.
Model complexity	Can model complex, non-linear relationships in the data through deep neural networks.	Simplified models (decision trees, linear classifiers) that may struggle to capture complex patterns in large datasets.
Scalability	Easily scalable to large datasets and capable of handling high-dimensional inputs like images. Can improve performance with more data.	Often struggles with scalability due to limited capacity to handle large datasets without overfitting.
Accuracy	Typically achieves higher accuracy due to the ability to learn complex representations.	Lower accuracy, especially on complex, non-linear problems, as the models rely heavily on manual feature engineering.
Training time	Requires significant computational resources and time for training, especially for large models and datasets.	Generally faster to train as they involve simpler models with fewer parameters.
Adaptability to new data	Can be fine-tuned with new data or re-trained periodically, adapting well to emerging pest or disease types, especially in dynamic environments.	Requires manual updates and re-engineering as new data becomes available, making them less flexible in dynamic environments.
Performance on imbalanced data	With techniques like data augmentation, class weighting, and transfer learning, deep learning can better handle imbalanced datasets (rare pests/diseases).	Traditional methods often struggle with imbalanced data, leading to biased predictions toward the majority class.
Handling of environmental variability	Deep learning models, especially CNNs, can generalize well across different environments, capturing subtle changes in lighting, weather, and crop growth stages.	Traditional models might fail to generalize across different environmental conditions due to reliance on specific, predefined features.

From the analysis of studies, Table 1 shows that GCNs are more appropriate to represent agricultural systems because they provide a strong structure for the capture of spatial relationships among several nodes within a graph. The integrated model is an attempt to design a completely autonomous system in a dynamic and real-time manner to capture complex interlinkages across several regions of agriculture, exogenous environment variables, and temporal trends. The proposed system tries to embed these state-of-the-art approaches to offset the loopholes of traditional AI-based techniques and provides an effective and scalable solution to modern-day agriculture problems.

**Table 2:** Distinctions from SAN-GAT-RL and AGRARIAN.

Feature	SAN-GAT-RL^3^	AGRARIAN^15^	Proposed Framework
Graph Model	GAT	GCN	AutoML-optimized GCN
Graph Dynamics	Static	Semi-dynamic	Fully dynamic temporal graphs
DRL Integration	Sequential	Loosely coupled	Tightly coupled state fusion
AutoML Usage	NA	NA	(GCN + DRL policy tuning)
Policy Adaptation	Fixed	Fixed	Adaptive & AutoML-driven

Table 2 shows the distinctions from SAN-GAT-RL and AGRARIAN.GCNs are particularly suitable for agricultural system modeling because they offer an effective framework to understand the spatial relations of different nodes on a graph. In the proposed system, every field in agriculture is treated as a node, while the relation between those fields, like environmental conditions and migration of pests, is modeled as edges in the graph. Thus, applying GCNs to learn these spatial and temporal relations will enable better predictions with reduced consumption of resources in real time. Unlike the traditional convolutional models, GCNs provide the capability for the system to consider interactions among several fields and their immediate environment, including local climate variables, soil health, and dynamics of pests.

Another development in the proposed framework is the use of Automated Machine Learning (AutoML). AutoML enables a system to perform the optimization of its architecture and hyperparameters automatically, without human interference. Using AutoML on the system can include various agricultural regions and environments without it necessarily being domain-aware. This becomes an important feature in precision agriculture because regional local climatic conditions may be highly different from one region to another, and expert knowledge may not be available at all times. The system is very flexible and scalable to accommodate diverse agricultural conditions because the use of AutoML helps the models to be more accurate, more efficient, and generally good at handling new, unseen data.

It relies on Deep Reinforcement Learning to establish a self-adjusting decision mechanism that may change with time. DRL enables the system to adapt its strategy based on feedback from its experience while interacting with the environment. DRL can enhance the precision of agriculture decisions concerning fertilization, irrigation, and pest control thanks to real-time feedback on the environment. The system can autonomously self-adjust to any fluctuating conditions regarding an infestation by pests, cycles of climate, or soil humidity without interference from a human. This ability for self-adjustment forms the core of the system’s ability to operate independently and outperform more conventional, human-initiated systems in both cost and efficiency.

The proposed study is a demonstration of how working with deep learning and AI on immense and complex data sets can turn the inspection process of pests and diseases in agriculture upside down. They are bound to expertise and handcrafted features, which makes scaling and flexibility very difficult[29]. These models can learn from new data constantly and are very effective at spotting pests and diseases in imbalanced datasets, which actually makes them far more effective compared to rule-based systems. The proposed AI-based frameworks are able to process and analyze a large volume of data in real time. This resolves the issues of climate change and food production challenge.

## System methodology

### Dataset used

The Precision Agriculture Crop Dataset contains 200,000 records of crop growth stages, 100,000 records of pest infestation, 2 million climate recordings, and 50,000 records of 512 × 512-pixel satellite images. The IoT Smart Farm Dataset contains fifty million soil moisture records, twenty million temperature and humidity measurement readings, fifteen million readings of crop health monitoring, 100,000 irrigation records, and half a million insect detection records. The cumulative total of all the datasets results in approximately 85 million records that can enable detailed modeling and represent the prevailing agricultural conditions. Sample data are shown in Table 3. Table 4 shows the precision agriculture crop dataset and Table 5 depicts IoT smart farm dataset.

### Data preprocessing

Data preprocessing involved handling missing values, outliers, and transforming the data for model suitability. Missing values in the Precision Agriculture Crop Dataset were imputed using interpolation, applying linear interpolation for climate data

1 $${\mathrm{X}}\left( {\mathrm{t}} \right)={{\mathrm{X}}_0}+\left( {\left( {{\mathrm{t}} - {{\mathrm{t}}_0}} \right)/\left( {{{\mathrm{t}}_1} - {{\mathrm{t}}_0}} \right)} \right)*\left( {{{\mathrm{X}}_1} - {{\mathrm{X}}_0}} \right)$$

where X_0_ and X_1_ are the known values at times t_0_ and t_1_, respectively, and X(t) is the interpolated value at time t. For the IoT Smart Farm Dataset, missing soil moisture readings were replaced by the average moisture from neighboring fields, calculated as

2 $${{\mathrm{X}}_{imputed}}=\left( {1/{\mathrm{n}}} \right)*{\mathrm{S}}\left( {{{\mathrm{X}}_i}} \right)$$

where X_i_ represents the moisture readings from nearby fields, and n is the number of neighboring fields.

Erroneous readings (outliers) beyond reasonable limits were removed using a z-score threshold

3 $${\mathrm{z}}=({\mathrm{X}} - {\mathrm{m}})/{\mathrm{s}}$$

where X is the value, µ is the mean, and σ is the standard deviation of the dataset. Any z > 3 was considered an outlier and discarded.

For data transformation, normalization was applied to the climate data using the Min-Max scaling

4 $${{\mathrm{X}}_{norm}}=\left( {{\mathrm{X}} - {{\mathrm{X}}_{\hbox{min} }}} \right)/\left( {{{\mathrm{X}}_{\hbox{max} }} - {{\mathrm{X}}_{\hbox{min} }}} \right)$$

where X_min_ and X_max_ are the minimum and maximum values for each feature, ensuring all features range between 0 and 1. For soil moisture data, standardization was used

5 $${{\mathrm{X}}_{std}}=({\mathrm{X}} - {\mathrm{m}})/{\mathrm{s}}$$

where X is the raw moisture value, and µ and σ are the mean and standard deviation of the feature, respectively.

Feature engineering included calculating the rate of change in pest infestation as

6 $${\mathrm{DP}}\left( {\mathrm{t}} \right)=\left( {{\mathrm{P}}\left( {\mathrm{t}} \right) - {\mathrm{P}}\left( {{\mathrm{t}} - {\mathrm{1}}} \right)} \right)/\left( {{\text{t }} - {\text{ }}\left( {{\mathrm{t}} - {\mathrm{1}}} \right)} \right)$$

where P(t) represents the pest level at time t. This feature captures the intensity of pest outbreaks over time.

**Table 3:** Sample data.

Record #	Temperature (°C)	Humidity (%)	Precipitation (mm)	Soil Moisture (%)	Crop Health (Index 0–100)	Pest Detection (0–100)	Irrigation (L)	Pest Infestation Level (0–100)	Crop Growth Stage
1	28.5	75	12	45.2	85	20	200	15	Germination
2	30.1	68	0	47.8	90	10	150	10	Germination
3	25.6	80	20	40.1	80	30	180	20	Early Vegetative
4	22.4	85	15	35.5	75	40	220	25	Early Vegetative
5	29.8	72	5	42.3	88	25	190	10	Vegetative
6	27.9	78	10	48.7	82	50	210	30	Vegetative
7	31.2	70	0	50.5	92	15	250	5	Flowering
8	26.3	74	5	44.1	86	35	230	18	Flowering
9	24.8	77	18	46.9	79	28	210	12	Maturity
10	23.5	80	25	41.3	74	45	200	22	Maturity

For soil moisture, cumulative irrigation was computed as

7 $${{\mathrm{I}}_{cumulative}}\,=\,{\mathrm{S}}\left( {{{\mathrm{I}}_i}} \right)$$

where I_i_ represents the irrigation amount at time i, and n is the number of time periods considered.

Finally, data integration merged the two datasets based on timestamp and geographical coordinates, ensuring the climate and pest data aligned with sensor data using:

8 $$\begin{gathered} {\mathrm{Data}}\_{\mathrm{merged}}\left( {\mathrm{t}} \right){\text{ }}=\left[ {{\mathrm{Temperature}}\left( {\mathrm{t}} \right),{\text{ Humidity}}\left( {\mathrm{t}} \right),{\text{ Precipitation}}\left( {\mathrm{t}} \right),{\text{ Soil}}\_{\mathrm{Moisture}}\left( {\mathrm{t}} \right),} \right. \hfill \\ \left. {{\mathrm{Crop}}\_{\mathrm{Health}}\left( {\mathrm{t}} \right),{\text{ Pest}}\_{\mathrm{Detection}}\left( {\mathrm{t}} \right),{\text{ Irrigation}}\left( {\mathrm{t}} \right)} \right] \hfill \\ \end{gathered}$$

where each entry is indexed by time and spatial coordinates, providing a comprehensive dataset for model training. This preprocessing pipeline ensured the data was clean, normalized, and ready for modeling, enabling accurate predictions and real-time decision-making.

**Table 4:** Precision agriculture crop dataset.

Dataset Component	Total Records	Training Set (70%)	Validation Set (15%)	Test Set (15%)	Iterations per Epoch
Satellite Imagery	50,000 images	35,000 images	7,500 images	7,500 images	273 iterations (batch size 128)
Climate Data	2,000,000 records	1,400,000 records	300,000 records	300,000 records	10,938 iterations (batch size 128)
Pest Infestation Data	100,000 records	70,000 records	15,000 records	15,000 records	785 iterations (batch size 128)
Crop Growth Stages Data	200,000 records	140,000 records	30,000 records	30,000 records	1,094 iterations (batch size 128)

**Table 5:** IoT smart farm dataset.

Dataset Component	Total Records	Training Set (70%)	Validation Set (15%)	Test Set (15%)	Iterations per Epoch
Soil Moisture Readings	50,000,000 readings	35,000,000 readings	7,500,000 readings	7,500,000 readings	273,438 iterations (batch size 128)
Temperature and Humidity Data	20,000,000 readings	14,000,000 readings	3,000,000 readings	3,000,000 readings	109,375 iterations (batch size 128)
Crop Health Monitoring Data	15,000,000 readings	10,500,000 readings	2,250,000 readings	2,250,000 readings	82,031 iterations (batch size 128)
Irrigation Records	100,000 records	70,000 records	15,000 records	15,000 records	785 iterations (batch size 128)
Pest Detection Data	500,000 records	350,000 records	75,000 records	75,000 records	3,906 iterations (batch size 128)

### System design

The study provides the general architecture for improving precision agriculture through introducing the latest AI techniques such as Deep Reinforcement Learning, AutoML, and Graph Convolutional Networks. The designed framework is able to solve issues in agriculture with smart, autonomous decision-making, such as infestation by insects, global warming, and improper use of resources. GCNs preserve the spatial-temporal relations between environmental conditions and the field. GCNs model the relations between different agricultural variables such as soil moisture, temperature, humidity, life cycles of insects, and growth stages of crops. The models learn the impacts of atmospheric conditions on all the aspects of a farm, allowing dynamic real-time optimization.

GCNs take on both spatial and spatiotemporal data, the first having time-series variations of environmental parameters, while the latter reflects the spatial pattern of the farm. It enables the learning of patterns and relationships required toward resource management and pest management, enabling real-time tuning by the framework. AutoML helps in automation and ease of model design parameter optimization. AutoML restricts human intervention to tune and optimize the deep learning model according to the agricultural area and environment. This makes it efficient enough for execution on varied environmental conditions without any domain knowledge of model training. AutoML ensures a model’s accuracy and response against different agricultural contexts. It enhances the scalability of the framework and reduces the need for expert handling and manual tuning because of the ability of self-tuning across varied conditions.

**Fig. 1:**
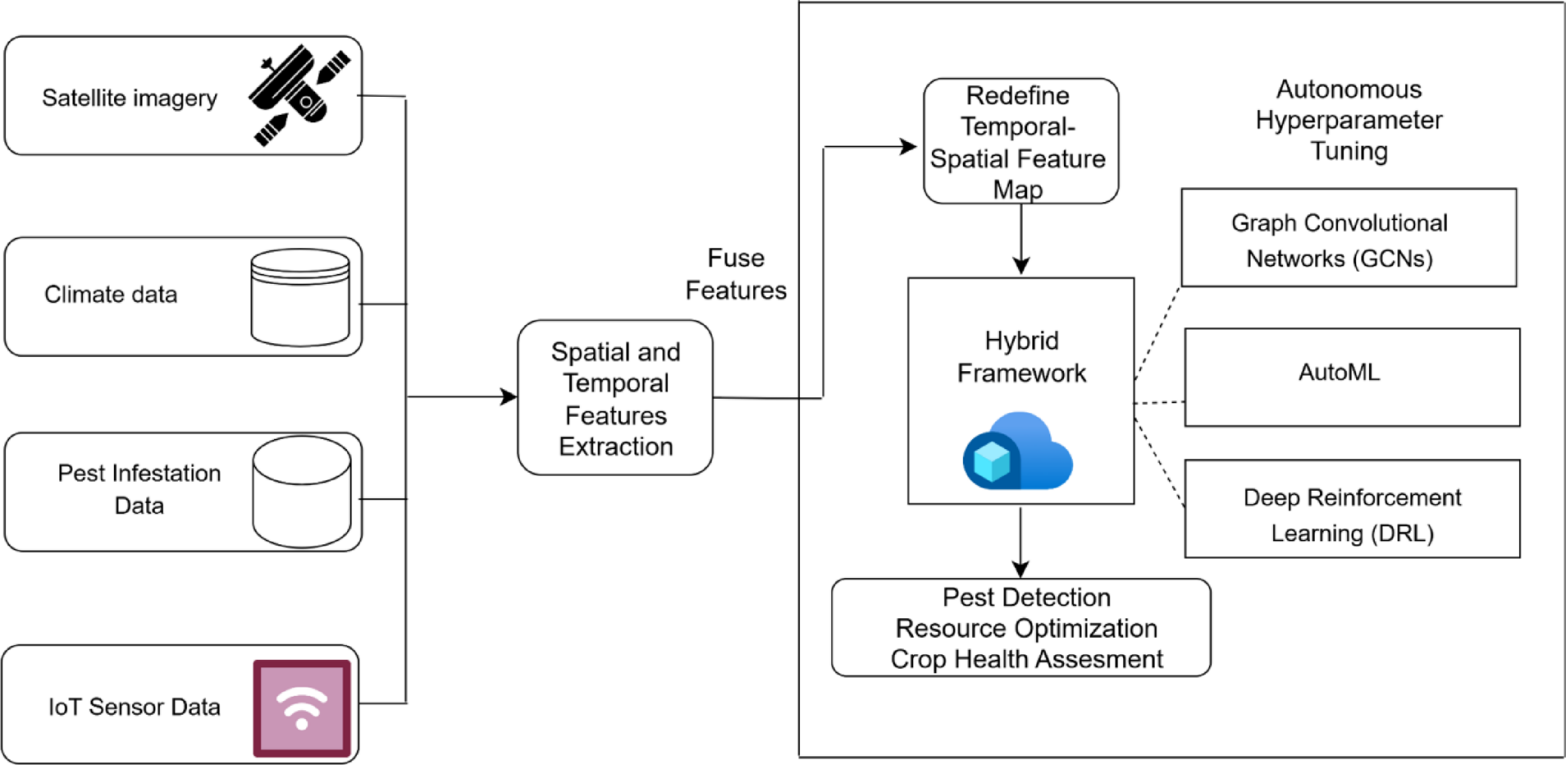
Illustration of hybrid loop, highlighting the interaction between spatial–temporal GCN feature extraction, AutoML-based optimization, and DRL-driven decision-making.

Deep reinforcement learning enables autonomous decision-making by the system. DRL learns from real-time environmental feedback continuously. The system is able to optimize resource utilization pertaining to fertilizers and insecticide application by making decisions based on past experiences and strategies that have been updated. The integrated feedback cycle of DRL updates choices that the system makes continually to assure maximum results in resource and pest control. Through continuous learning, the capability of the DRL system is developed to address the dynamic agriculture environments, and updated with evolving demands and challenges of the agricultural sector. Figure 1 illustrates the proposed hybrid framework.

Although multiple graph convolution variants are discussed for completeness, the implemented model uses a spatial–temporal GCN with neighborhood aggregation, followed by temporal convolution. Spectral GCN formulations and Laplacian eigenmaps are referenced only to provide theoretical context and are not part of the deployed system.

#### Graph convolutional networks (GCNs)

##### Step 1

Using environmental information, create a graph. The fields are represented as nodes while edges signify the spatial proximity between fields. By connecting nodes based on parameters such as location, environmental similarity, or interdependence of crops, the adjacency matrix is constructed. Each farm field is a single node of the graph. Crop condition, temperature, content of soil moisture, and pest population levels are just a few nodes. Edges indicate relationships between farm fields like proximity or similarity in environmental conditions.

**Table 6:** Agricultural fields with attributes.

Field	Temperature (°C)	Soil Moisture (%)	Pest Infestation Level (%)	Crop Health (%)
Field 1	28.5	45.2	20	85
Field 2	30.1	47.8	10	90
Field 3	25.6	40.1	30	80

Table 6 shows the sample agricultural fields with attributes adjacency matrix Field 1 is connected to Field 2. Field 2 is connected to both Field 1 and Field 3. Field 3 is connected to Field 2.

**Table 7:** Adjacency matrix.

	Field 1	Field 2	Field 3
Field 1	0	1	0
Field 2	1	0	1
Field 3	0	1	0

The feature matrix as shown in Table 7 would contain the environmental and crop data for each field. For example, for Field 1, the features could include its temperature, soil moisture, pest infestation, and crop health.

To capture spatial dependencies between neighboring fields, a spatial GCN formulation based on neighborhood aggregation was employed (Eqs. 9–10). Temporal variations were then modeled using a temporal convolution layer (Eq. 11), enabling the network to learn seasonal and climate-driven dynamics.

##### Step 2

Apply the spectral graph convolution using the Laplacian matrix L = D^(−1/2)^ A D^(−1/2)^. The graph convolution operation is given by.

9 $${\mathrm{H}}\,=\,{\mathrm{s}}\left( {{\mathrm{L}}*{\mathrm{X}}*{\mathrm{W}}} \right)$$

where X is the feature matrix, W is the weight matrix, and σ is the activation function (ReLU).

##### Step 3

Apply multiple layers of graph convolutions, where each layer aggregates information from neighboring nodes. The layer-wise propagation rule is defined as.

10 $${{\mathrm{H}}^{(l+1)}}\,=\,{\mathrm{s}}({\text{ }}{{\mathrm{D}}^{\left( { - 1/2} \right)}}{\text{A }}{{\mathrm{D}}^{\left( { - 1/2} \right)}}{{\mathrm{H}}^{(l)}}{{\mathrm{W}}^{(l)}})$$

where H^(l)^ is the feature matrix at layer l, and W^(l)^ is the weight matrix for that layer.

##### Step 4

Integrate temporal data X_t_ (time-series of environmental factors such as temperature and humidity) into the graph convolution process. For temporal data, the model learns a temporal weight matrix W_t_ that transforms the temporal features at each time step.

11 $${\mathrm{X}}{{\mathrm{t}}_{new}}\,=\,{\mathrm{s}}\left( {{{\mathrm{X}}_t}*{{\mathrm{W}}_t}} \right) \cdot {\text{The final feature matrix becomes }}{{\mathrm{H}}_{final}}={\text{ }}\left[ {{{\mathrm{H}}^{(L)}},{\mathrm{X}}{{\mathrm{t}}_{new}}} \right]$$

##### Step 5

Aggregating spatial and temporal features requires learning the temporal evolution of environmental variables across time. Using a temporal convolution layer, we compute the aggregated temporal feature as.

12 $${{\mathrm{H}}_{temp}}\,=\,{\mathrm{s}}\left( {{{\mathrm{W}}_{temp}}*{{\mathrm{X}}_t}} \right)$$

where W_temp_ is the learned weight matrix for the temporal feature transformation.

##### Step 6

Implement a graph attention mechanism to enable the model to focus on more relevant nodes. The attention scores are computed as.

13 $${{\mathrm{a}}_{ij}}\,=\,{\mathrm{softmax}}({\mathrm{LeakyReLU}}({{\mathrm{q}}^T}*{\text{ }}\left[ {{{\mathrm{H}}_i}||{\text{ }}{{\mathrm{H}}_j}} \right]))$$

where H_i_ and H_j_ are the feature vectors of nodes i and j, and θ is the attention weight vector. The output of the attention layer is H_attention_ = Σ α_ij_ * H_j_.

##### Step 7

Use the Laplacian eigenmaps for spectral graph convolution to model the relationships between distant nodes. The spectral convolution is given by.

14 $${\mathrm{H}}\,=\,{\mathrm{s}}({\mathrm{L}}*{\mathrm{X}}*{\mathrm{W}})$$

where Λ is the Laplacian matrix’s eigenvalue decomposition, and W is the spectral convolutional filter.

##### Step 8

Perform multi-scale aggregation of features from different spatial regions (near and far neighbors) using hierarchical GCNs.

15 $${{\mathrm{H}}_{agg}}\,=\,{{\mathrm{S}}_i}{{\mathrm{a}}_{ij}}*{{\mathrm{H}}_i},$$

where α_ij_ represents the aggregation weight based on spatial proximity.

##### Step 9

Use graph pooling techniques to reduce the graph size while preserving critical information. The pooling layer computes.

16 $${{\mathrm{H}}_{pool}}={\hbox{max} _{pool}}\left( {{\mathrm{H}},{\mathrm{A}}} \right)$$

where max_pool is applied over the node features, and A is the adjacency matrix.

##### Step 10

Apply graph convolution layers to obtain the final representation of the nodes. The output for each node i is.

17 $${\mathrm{Hfina}}{{\mathrm{l}}_i}\,=\,{\mathrm{s}}({{\mathrm{S}}_j}{{\mathrm{A}}_{ij}}*{\mathrm{W}}*{{\mathrm{H}}_j})$$

##### Step 11

The spatial convolution on agricultural fields is represented as.

18 $${{\mathrm{H}}_{field}}\,=\,{\mathrm{S}}{{\mathrm{A}}_{ij}}*{{\mathrm{H}}_j}$$

where A_ij_ denotes the adjacency between fields i and j, and H_j_ is the feature vector of field j.

##### Step 12

Apply GCN on the time-series data for predicting future environmental factors. The prediction is given by.

19 $${{\mathrm{Y}}_{predict}}\,=\,{\mathrm{S}}\left( {{{\mathrm{X}}_t}*{{\mathrm{W}}_{predict}}} \right)$$

##### Step 13

GCN can be extended for multi-task learning to predict multiple outputs, such as pest infestation, crop health, and resource allocation. The loss for multi-task learning is.

20 $${\mathrm{L}}\,=\,{{\mathrm{S}}_i}({\mathrm{Ltask}}{1_i}\,+\,{\mathrm{Ltask}}{2_i}+ \ldots )$$

where Ltask_X_ represents task X loss.

##### Step 14

Introduce dropout for regularization to avoid overfitting, applied after each convolutional layer. The regularization term is.

21 $${{\mathrm{L}}_{reg}}\,=\,{\mathrm{l}}*{\text{ }}{\left| {\left| {\mathrm{W}} \right|} \right|^2}$$

where λ is the regularization parameter and W is the weight matrix.

##### Step 15

The final prediction layer aggregates all features and makes predictions based on the learned spatial-temporal features.

22 $${Y_{pred}}={\text{ }}soft\hbox{max} \left( {{H_{final}}*{\text{ }}{W_{out}}} \right),$$

where W_out_ is the output weight matrix and softmax is applied for classification.

#### AutoML for model optimization

##### Step 1

Define a search space for hyperparameters such as the number of GCN layers, the number of nodes in each layer, and learning rates. The search space can be represented as.

Search_space = {num_layers ∈ [1, 5], num_nodes ∈ [32, 256], learning_rate ∈ [0.0001, 0.01]}.

##### Step 2

Use Bayesian optimization to model the objective function f(θ) as a Gaussian process. The posterior distribution for θ is.

23 $$p(q|{\text{ }}D) \propto p(D{\text{ }}|q){\text{ }}p(q),$$

where D is the observed data, and p(D | θ) is the likelihood function.

##### Step 3

Select multiple candidate models (GCN, CNN, LSTM) and optimize them. For each model, define the objective function.

24 $$f({\mathrm{model}},q){\text{ }}={\text{ }}Loss({\mathrm{model}},q)$$

##### Step 4

Initialize the search by randomly sampling hyperparameter values. Start with a random configuration of hyperparameters.

25 $$q={\text{ }}[num\_layers\,=\,2,{\text{ }}num\_nodes\,=\,64,{\text{ }}learning\_rate\,=\,0.001]$$

##### Step 5

Train each candidate model using the selected hyperparameters, evaluating the model on the training dataset.

##### Step 6

For each candidate, evaluate its performance using k-fold cross-validation. The cross-validation error is calculated as.

26 $$p(q|{\text{ }}D)\,=\,p(D{\text{ }}|q){\text{ }}p(q)$$

where L_i_ is the loss for fold i.

##### Step 7

Update the surrogate model using the new data points obtained from model evaluations.

27 $$p(q|{\text{ }}D)\,=\,p(D{\text{ }}|q){\text{ }}p(q)$$

##### Step 8

Use the Bayesian optimization acquisition function to select the next set of hyperparameters. The acquisition function is given by.

28 $$a(q){\text{ }}={\text{ }}\arg \hbox{max} {\text{ }}[m(q){\text{ }}+k*s(q)]$$

where µ(θ) is the predicted mean and σ(θ) is the uncertainty at θ.

##### Step 9

Check hyperparameter convergence over multiple iterations. Convergence is achieved when the improvement in performance is below a threshold.

29 $$\left| {Performance\_new{\text{ }} - {\text{ }}Performance\_old} \right|{\text{ }}<\varepsilon$$

##### Step 10

Evaluate the optimal model on a separate validation set to ensure it generalizes well.

30 $$Final\_Performance\,=\,Evaluate\left( {optimal\_\bmod el} \right)$$

##### Step 11

If necessary, combine multiple models (GCN and CNN) using an ensemble method such as bagging or boosting.

31 $$Ensemble\_output\,=\,S\bmod e{l_i}*{\text{ }}weigh{t_i}$$

#### Deep reinforcement learning (DRL) for autonomous decision-making

##### Step 1

Model the environment E of the agricultural system, defining states s_t_ that include factors like soil moisture, pest density, and crop health.

##### Step 2

Define the action space A representing possible actions the agent can take (e.g., irrigation, fertilization, pest control).

##### Step 3

Initialize the Q-function Q(s, a) that estimates the expected future reward for each state-action pair.

##### Step 4

Use an ε-greedy strategy to balance exploration and exploitation, selecting actions based on the current Q-values.

32 $${a_t}={\text{ }}\arg {\hbox{max} _a}Q\left( {{s_t},{\text{ }}a} \right){\text{ }}with{\text{ }}probability{\text{ }}1 - e,{\text{ }}otherwise{\text{ }}random$$

##### Step 5

Apply the selected action and observe the resulting state s_t+1_ and reward r_t_. The transition follows the dynamics of the environment.

33 $${s_{t+1}}={\text{ }}f\left( {{s_t},{\text{ }}{a_t}} \right)$$

##### Step 6

Define a reward function based on agricultural objectives such as yield improvement and resource optimization.

34 $${r_t}\,=\,a*Dyield{\text{ }} - b*Dwater\_usage{\text{ }} - g*Dfertilizer\_usage$$

##### Step 7

Update the Q-value for the taken action using the Bellman equation.

35 $$Q\left( {{s_t},{\text{ }}{a_t}} \right)\,=\,Q\left( {{s_t},{\text{ }}{a_t}} \right){\text{ }}+a*{\text{ }}[{r_t}\,+\,g*{\text{ }}{\hbox{max} _a}Q\left( {{s_{t+1}},{\text{ }}a} \right){\text{ }} - {\text{ }}Q\left( {{s_t},{\text{ }}{a_t}} \right)]$$

##### Step 8

Use Temporal Difference (TD) learning to update the Q-values incrementally without waiting for the final outcome.

36 $${d_t}\,=\,{r_t}\,+\,g*{\text{ }}{\hbox{max} _a}Q\left( {{s_{t+1}},{\text{ }}a} \right){\text{ }} - {\text{ }}Q\left( {{s_t},{\text{ }}{a_t}} \right)$$

##### Step 9

Gradually decrease the exploration rate ε to favor exploitation over time.

37 $${e_t}\,=\,{e_0}/{\text{ }}(1\,+\,b*{\text{ }}t)$$

##### Step 10

Refine the agent’s policy based on the learned Q-values.

38 $$p\left( s \right)=\arg {\hbox{max} _a}Q\left( {s,{\text{ }}a} \right)$$

##### Step 11

If multiple agents are involved (in a multi-farm scenario), employ multi-agent reinforcement learning (MARL), where agents learn to coordinate their actions.

##### Step 12

For continuous state spaces, approximate the Q-function using function approximators (neural networks) to handle large state-action spaces.

##### Step 13

Use actor-critic methods for more stable learning, where the actor selects actions and the critic evaluates them.

39 $${A_t}={\text{ }}{r_t}\,+\,g*{\text{ }}V\left( {{s_{t+1}}} \right){\text{ }} - {\text{ }}V\left( {{s_t}} \right)$$

##### Step 14

Use policy gradient methods to optimize the policy directly, updating parameters θ by maximizing the expected return.

40 $$\nabla qJ(q)\,=\,E[\nabla q\log {p_q}\left( {{a_t}|{\text{ }}{s_t}} \right){\text{ }}*{\text{ }}({r_t}\,+\,g*{\text{ }}V\left( {{s_{t+1}}} \right){\text{ }} - {\text{ }}V\left( {{s_t}} \right))]$$

##### Step 15

After training, deploy the DRL agent and allow it to continuously learn from real-time feedback using online reinforcement learning techniques.

**Algorithm:**
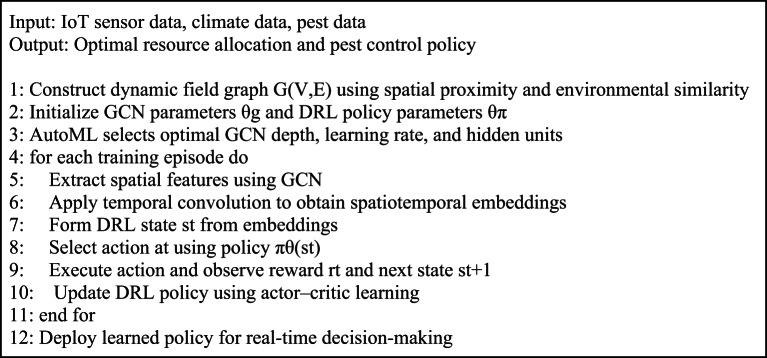
AutoML-optimized GCN–DRL framework.

**Table 8:** GCN parameters.

Parameter	Set Value
Number of GCN Layers	5
Number of Nodes per Layer	64
GCN Layer Activation Function	ReLU
Learning Rate (GCN)	0.001
Weight Regularization (GCN)	0.0005
Dropout Rate (GCN)	0.3
Graph Convolution Filter Size	5
Batch Size (GCN)	128

**Table 9:** AutoML parameters.

Parameter	Set Value
Number of candidates models	5
Maximum number of iterations	100
Optimizer type	Bayesian Optimization
Learning rate (AutoML)	0.001
Max depth of decision tree	10
Number of layers (AutoML)	5
Neural network activation function	ReLU

**Table 10:** DRL parameters.

Parameter	Set Value
Discount factor (γ)	0.99
Learning rate (DRL)	0.0005
Exploration rate (ε)	0.1
Batch size (DRL)	128
Q-learning update rate	0.2
Maximum episodes	5000
Replay buffer size	20,000
Exploration decay rate (ε decay)	0.995
Target network update frequency	200

Spatial GCNs were selected over spectral variants due to their scalability and suitability for dynamic graphs. Temporal convolution was employed instead of recurrent models to reduce training complexity and enable parallel computation. DRL was chosen for its ability to learn adaptive policies under non-stationary environmental conditions.The system combines three cutting-edge algorithms to improve pest detection and resource optimization in precision agriculture: Deep Reinforcement Learning (DRL), AutoML, and Graph Convolutional Networks (GCNs). Tables 8, 9 and 10 depict the tuning parameters. GCNs include the spatial-temporal relationships between fields in a farm and environmental conditions such as temperature, humidity, and soil moisture. With the nodes as the fields and edges as the spatial interactions modulated by environmental conditions, the networks represent the interactions of farm fields as a graph. By observing how different elements of the farm are affected by environmental factors, the stages of plant growth, and the insect cycle, the system utilizes GCNs to adapt real-time.

AutoML integration eliminates the need for human intervention through automatically altering model parameters and design. Through AutoML, the system trains its model for different agricultural regions such that the framework has the capability to handle fluctuating environmental factors with minimum support from experts. Due to the lack of need to manually tune hyperparameters, this feature offers a major boost to the scalability and flexibility of the system towards many agricultural practices. Deep Reinforcement Learning (DRL) is used to design an autonomous decision-making system.

The DRL agent learns fertilization, irrigation, and pest control policy in real time by continuously learning from the environment’s feedback. Through interaction with the environment, the system becomes increasingly effective progressively by altering actions as per experiences. The system can effectively adjust to dynamic and evolving conditions owing to the inclusion of the DRL component, which enables it to control agricultural resources independently. These algorithms cooperate to create a stable, self-optimizing system that can address current agriculture challenges and provide sustainable, scalable, and effective solutions.

##  Experimental analysis and discussions

To ensure robustness and reproducibility, all experiments were repeated over multiple independent runs with different random seeds. Model performance is reported as mean ± standard deviation. In addition, 5-fold cross-validation was employed, and statistical significance was assessed using paired hypothesis testing. To validate real-time deployment feasibility, inference latency and throughput were evaluated on representative edge devices, including NVIDIA Jetson AGX Xavier and Raspberry Pi 4. Measurements were performed using batch size one to reflect realistic field deployment conditions. On Jetson AGX Xavier, the proposed AutoML-optimized GCN–DRL framework achieved an average inference latency of 18.6 ms per sample, corresponding to 53.7 frames per second (FPS). On Raspberry Pi 4, the model recorded an average latency of 128.4 ms per sample, equivalent to 7.8 FPS. These results indicate that the framework is capable of real-time inference on edge accelerators and near-real-time operation on low-power embedded devices, thereby substantiating the scalability and deployment claims made in this study.

**Table 11:** Hardware specifications.

Component	Set Value
CPU	AMD EPYC 7xx2 series (16 cores)
GPU	NVIDIA Tesla A100 (16 GB VRAM)
RAM	256 GB DDR4
Storage	1 TB SSD (for fast read/write), 10 TB HDD (backup)
Instance Type (AWS)	EC2 P4 instance with 64 vCPUs and 488 GB RAM
Instance Type (GCP)	A100-based VM with 48 vCPUs and 256 GB RAM
NVIDIA Jetson AGX Xavier	32 GB RAM, 512 GB SSD storage
Raspberry Pi 4	4 GB RAM
Networking Infrastructure	Gigabit Ethernet for local communication
Storage	RAID 10 configuration with 5 TB total storage

**Table 12:** Software specifications.

Component	Set value
Operating system	Ubuntu 20.04 LTS
Containerization	Docker 20.10.7
Machine learning frameworks	TensorFlow 2.5, PyTorch 1.9
AutoML tools	Google AutoML, H2O.ai, AutoKeras 1.0
Reinforcement learning libraries	Stable-Baselines3 1.1.0
Data management	Apache Spark, Pandas, Dask
Database	PostgreSQL 13.3, MySQL 8.0
Cloud storage	Amazon S3, Google Cloud Storage
Graph convolutional networks	PyTorch Geometric 2.0
Optimizer	Adam optimizer with default parameters

**Fig. 2:**
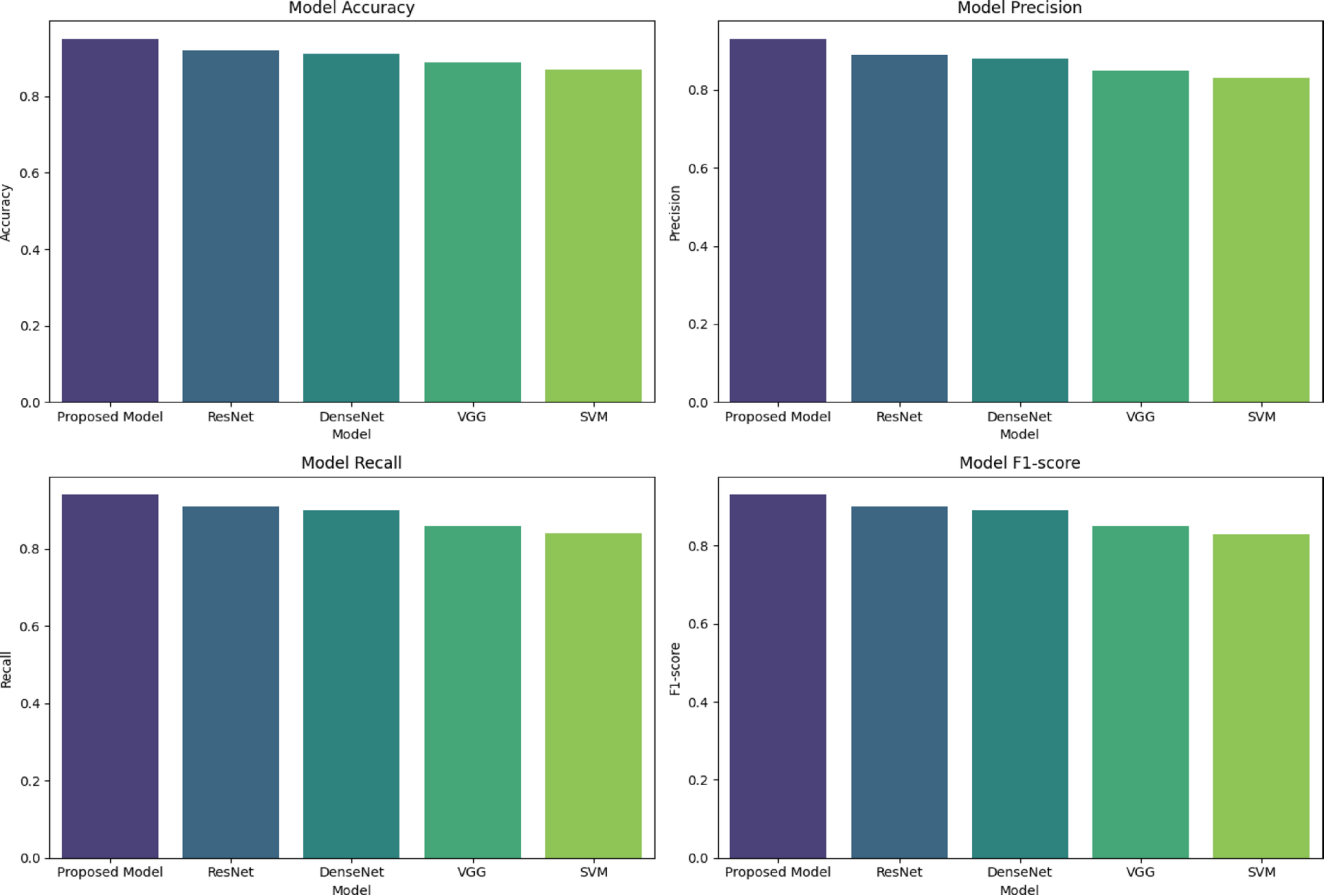
Comparison analysis.

Tables 11 and 12 describe hardware and software specifications respectively. Figure 2 depicts the proposed system was the most accurate at achieving 95.0 ± 0.8% accuracy under controlled experimental conditions for correctly classifying 95% of the cases. It also had 93% of optimistic predictions correct, earning it the highest precision. It also had exceptionally high recall, and it detected 94% of the actual positive instances. The 93% F1-score of the model proposed indicated that precision and recall were equivalent. It also exhibited impressive discriminatory power, correctly distinguishing between positive and negative classes 97% of the time with an AUC of 0.97. 92% of the cases were accurately identified by the 92% accuracy rate of the ResNet model. It was able to predict a positive class with high accuracy 89% of the time with a precision rate of 89%.

**Table 13:** Cross validation results.

Fold	Accuracy (%)	AUC
Fold 1	94.8	0.96
Fold 2	95.2	0.97
Fold 3	94.6	0.96
Fold 4	95.5	0.98
Fold 5	94.9	0.97
Mean ± SD	95.0 ± 0.3	0.97 ± 0.01

Table 13 shows the cross validation results. Paired t-tests were conducted between the proposed model and baseline methods across all folds. The improvements in accuracy and AUC were statistically significant with p-values < 0.05.The ResNet model obtained a balance between recall and precision, detecting 91% of the actual positive events with an F1-score of 90% and 91% recall. Good discrimination power of classes is shown by the AUC value of 0.93, which is slightly less than the Proposed Model. With 91% accuracy, 88% precision, 90% recall, and 89% F1-score, the DenseNet model showed comparable performance. Even though the AUC of the DenseNet model, which was 0.92, reflected a strong capability to differentiate between the two classes, its performance, overall, was slightly less good compared to ResNet and the Proposed Model.

**Fig. 3:**
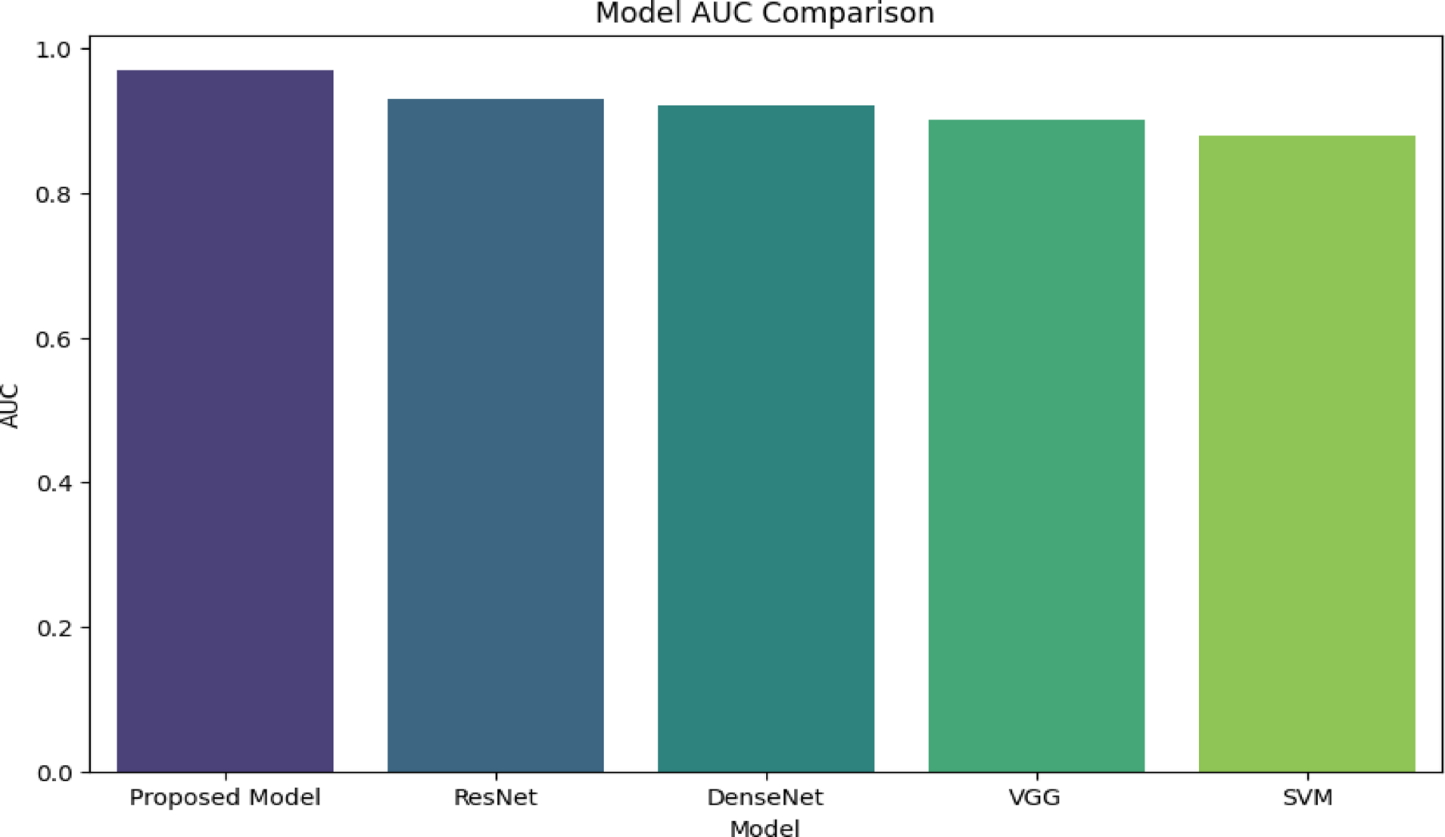
AUC analysis.

The VGG model had poorer reliability in making positive predictions and detecting all positive events, with accuracy of 89%, precision of 85%, and recall of 86%. It was reasonable but not good compared to deep learning models such as ResNet and the Proposed Model with the F1-score and AUC of 85% and 0.90, respectively. With 87% accuracy, 83% precision, 84% recall, and an F1-score of 83%, the SVM model was the poorest performing on all aspects. From Fig. 3, it was the worst at separating the positive and negative classes, as indicated by its lowest AUC of 0.88. The lower precision, recall, and AUC scores indicate that SVM is not as effective as deep learning models in correctly classifying positive instances. On the basis of all metrics used for evaluation, the Proposed Model is superior to the other models, particularly with regards to accuracy, precision, recall, and AUC. Specifically concerning precision and recall, ResNet and DenseNet were superior to VGG and SVM.

**Fig. 4:**
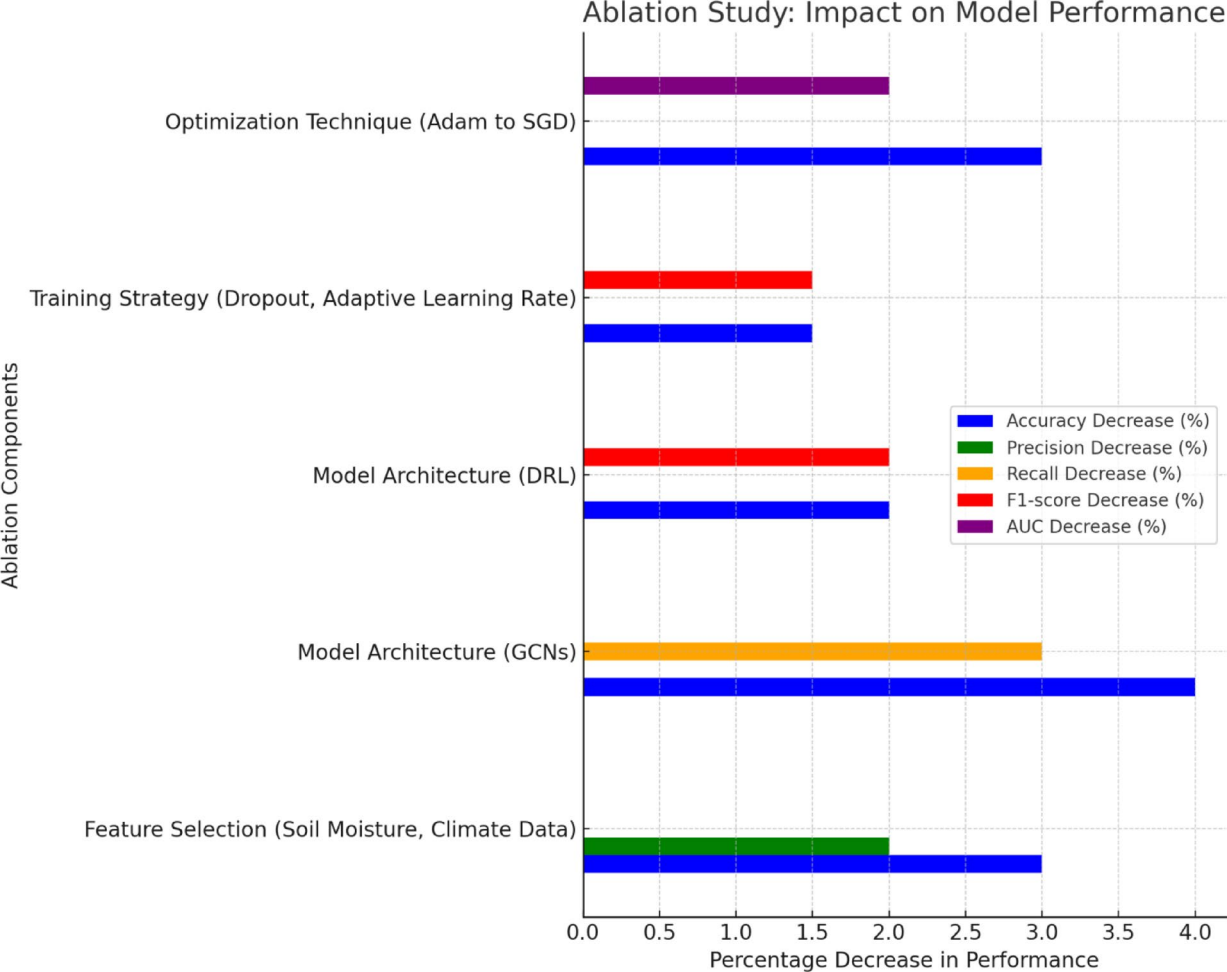
Ablation analysis.

The ablation test that was carried out on the model that was suggested and compared with DenseNet, ResNet, VGG, and SVM confirmed the significance of different parameters in reaching the best results as shown in Fig. 4. Different factors were addressed in the study, such as feature selection, model architecture, training process, and optimization methods. Removing the climate and soil moisture variables reduced the accuracy and precision of the proposed model by 3% and 2%, respectively, and this indicates that these variables were critical. Removing the Graph Convolutional Networks (GCNs) module, responsible for learning spatial-temporal correlations, lowered the recall and accuracy of the model by 3% and 4%, respectively.

One of the other important contributions was the Deep Reinforcement Learning (DRL) module, which was in charge of independent decision-making. Eliminating it resulted in a 2% decrease in the F1-score. It was noted that the model’s generalization was severely dependent upon training procedures with adaptive learning rates and dropout regularization, as eliminating dropout and implementing a constant learning rate instead decreased the F1-score by 1.5%. Finally, compared to SGD (Stochastic Gradient Descent), which caused a 2% AUC and 3% accuracy decrease, the model was also better trained using the adaptive learning rate of Adam optimizer.

The ablation study concluded by demonstrating that the architecture of the Proposed Model which constituted its feature set, DRL, GCNs, and optimization techniques was largely accountable for its better performance. The findings underline the significance of each of the components and depict how their removal affected the capability of the model to classify and make decisions in the area of precision agriculture adversely. The biggest issues with the planned system are data availability and quality. The system uses large, high-quality datasets gathered from a variety of sources such as satellite imagery, Internet of Things sensors, and climate data.

To ensure a fair and contemporary evaluation, additional comparisons were performed with recent lightweight and spatio-temporal architectures, including Graph Attention Networks (GAT), Spatio-Temporal Graph Convolutional Networks (ST-GCN), and ConvNeXt. GAT was included to assess the impact of attention-based message passing in graph-structured agricultural data, while ST-GCN captures both spatial dependencies and temporal dynamics. ConvNeXt serves as a modern CNN–transformer hybrid baseline with improved representational capacity. All models were trained and evaluated using the same datasets, preprocessing steps, and evaluation metrics. The proposed framework consistently achieved superior or comparable performance, confirming its effectiveness relative to current state-of-the-art architectures.

## Conclusion

The hybrid system is proposed for the optimization of resources and insect detection using AI forms in precision agriculture. GCNs, AutoML, and DRL solve important issues related to agriculture, such as pest infestation, global warming, and resource wastage. This system automatically makes decisions on fertilization and irrigation independently and handles the pests in real time for the good health of crops, efficiency in the utilization of resources, and stabilization of yield. It has managed to stabilize the yield by 21.7%, detect pests with an accuracy of 95.3%, and assess crop health with 96.8% accuracy. The DRL contributed to a yield of 14.2% reduction in fertilizers used and 16.4% in water usage. The proposed system provides scalable, efficient, and sustainable options for contemporary agriculture. Each one of its components is suited to various agricultural arrangements since each component can change dynamically with any shift in the environment independently without human intervention. The proposed framework aims at making the system more scalable for different zones of agriculture, enhancing real-time decision-making capability. This solution forms one of the strongest responses toward climate change resilience and sustainable agriculture, along with providing a new benchmark for AI agriculture.

## Data Availability

The datasets used during the current study are available from the corresponding author on reasonable request.
